# Thiazolidinedione use is associated with reduced risk of dementia in patients with type 2 diabetes mellitus: A retrospective cohort study

**DOI:** 10.1111/1753-0407.13352

**Published:** 2023-01-20

**Authors:** Houyu Zhao, Lin Zhuo, Yexiang Sun, Peng Shen, Hongbo Lin, Siyan Zhan

**Affiliations:** ^1^ Department of Epidemiology and Biostatistics, School of Public Health Peking University Beijing China; ^2^ Research Center of Clinical Epidemiology Peking University Third Hospital Beijing China; ^3^ Yinzhou District Center for Disease Control and Prevention Ningbo China; ^4^ Center for Intelligent Public Health, Institute for Artificial Intelligence Peking University Beijing China

**Keywords:** dementia, population‐based cohort, thiazolidinediones, type 2 diabetes mellitus, 噻唑烷二酮类药物, 痴呆症, 2型糖尿病, 基于人群的队列研究。

## Abstract

**Background:**

Type 2 diabetes mellitus (T2DM) and dementia cause heavy health burden in mainland China, where few studies have investigated the association between glucose‐lowering agents and dementia risk. We aimed to assess the association between use of thiazolidinediones (TZDs) and dementia incidence in a mainland Chinese population with T2DM.

**Methods:**

A retrospective cohort of T2DM patients who were new users of TZDs or alpha glucosidase inhibitors (AGIs) was assembled using the Yinzhou Regional Health Care Database. A Cox model with inverse probability of treatment weighting (IPTW) for controlling potential founding was applied to estimate the hazard ratio (HR) of the association between use of TZDs and dementia risk.

**Results:**

A total of 49 823 new users of AGIs and 12 752 new users of TZDs were included in the final cohort. In the primary analysis, the incidence of dementia was 195.7 and 78.2 per 100 000 person‐years in users of AGIs and TZDs respectively. TZD use was associated with a reduced risk of incident dementia after adjusting for potential confounding using IPTW, with a HR of 0.51 (95% CI, 0.38–0.67). The results in various subgroup analyses and sensitivity analyses were consistent with the findings of the primary analysis.

**Conclusions:**

Use of TZDs is associated with a decreased risk of dementia incidence in a mainland Chinese population with T2DM.

## Graphical abstract

**Highlights**

- Type 2 diabetes mellitus (T2DM) and dementia cause heavy health burden in China.
- This was the first cohort study investigating the association between TZD use and dementia incidence in a mainland Chinese population.
- We found that TZD use was associated with a 49% reduction in incidence of dementia.
- Our results could provide some insights into developing effective prevention and control measures to reduce the future disease burden of dementia.

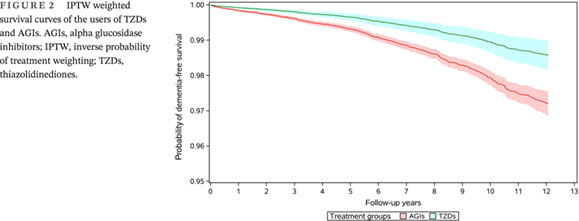

## 摘要

**背景:**2型糖尿病(T2DM)和痴呆给患者带来严重的健康负担，但目前很少有研究探讨降糖药物与痴呆风险之间的关系。我们旨在评估T2DM人群中噻唑烷二酮类药物的使用与痴呆发病率之间的关系。

**研究设计和方法:**利用鄞州地区卫生保健数据库，对新使用噻唑烷二酮(TZDs)或α‐糖苷酶抑制剂(AGIs)的2型糖尿病患者进行回顾性队列研究。采用治疗加权逆概率(IPTW)控制潜在发病的Cox模型来估计TZD使用与痴呆风险之间的关联的风险比(HR)。

**结果:**最终队列共纳入49823名AGIs新用户和12752名TZDs新用户。在初步分析中，AGIs和TZDs使用者的痴呆发病率分别为每10万人195.7/年和78.2/年。在使用IPTW进行潜在混杂调整后，TZDs的使用与痴呆事件风险降低相关，HR为0.51(95%CI,0.38‐0.67)。不同亚组分析和敏感性分析的结果与初步分析的结果一致。

**结论:**在2型糖尿病人群中，使用TZDs与痴呆发病率降低相关。

## 1 INTRODUCTION

Type 2 diabetes mellitus (T2DM) and dementia are two major global health challenges, with over 460 million and more than 55 million people living with these diseases respectively.^1^, ^2^, ^3^ With the diabetes pandemic and increase of aging population worldwide, the incidence and disease burden of dementia are expected to increase.^4^ Growing evidence from epidemiological and preclinical studies has suggested that T2DM and dementia share many similar pathological mechanisms,^5^ among which insulin resistance and resulting hyperinsulinemia are two main characteristics of T2DM, contributing to a higher risk of dementia in elderly people.^5^ In view of their common pathogenesis, and given the lack of effective treatment for dementia, repositioning and repurposing antidiabetic drugs for preventing or treating dementia have attracted accumulating attention in recent years.^6^, ^7^, ^8^

Thiazolidinediones (TZDs) are a class of insulin sensitizer, reducing insulin resistance through stimulating peroxisome proliferator‐activated receptor gamma (PPARγ),^9^ which can regulate protein carbohydrate, lipid metabolism, and inflammatory responses, making TZDs a potential treatment for insulin resistance in the brain.^9^, ^10^ Several epidemiological studies have assessed the association between TZD use and risk of dementia, with inconsistent results, which may be due to the methodology issues, including confounding by indication and time‐related bias^11^ arising when comparing ever user of TZDs with untreated patients or users of antidiabetic agents that are prescribed in different stages of T2DM,^12^, ^13^, ^14^, ^15^, ^16^, ^17^ as well as residual confounding caused by uncontrolled confounders of lifestyle information and disease severity of diabetes.^12^, ^16^, ^18^, ^19^ Moreover, no real‐word study of the association between the use of TZDs and dementia incidence has been reported in mainland China, where approximately 24% of global dementia patients live.^2^, ^20^ In this background, we conducted a retrospective population‐based cohort study applying an active‐comparator new‐user (ACNU) design to evaluate the association between TZD use and risk of dementia in a Chinese population with T2DM.

## 2 MATERIALS AND METHODS

### 2.1 Data source and patients

We assembled a retrospective cohort of new users of TZDs and alpha glucosidase inhibitors (AGIs) using the Yinzhou Regional Health Care Database (YRHCD). The YRHCD integrated longitudinal information of electronic medical records, disease registry and management, death registry, and other healthcare services in the Yinzhou District, Ningbo City of China.^21^, ^22^ Diabetes patients were registered in the disease registry system and would be followed up quarterly by community physicians, with common health measures, such as c(FPG) and glycated hemoglobin (HbA1c), being measured or asked.^22^ T2DM patients were identified by linking the disease registry system and electronic diagnosis records, if they (a) were registered in the diabetes registry system and diagnosed with T2DM; or (b) had more than two diagnosis records of T2DM and no records of type 1 diabetes in the electronic medical records. Information of drug exposure, covariates, and outcome was extracted through linking longitudinal records of drug prescription, laboratory examination, and outpatient and inpatient visits. The data used in this study and their relationship were presented in Figure S1 and have been outlined in detail previously.^22^

AGIs were chosen as the comparator because TZDs and AGIs are two classes of second‐line oral glucose lowering agents commonly used at the same stage of T2DM in China.^23^ AGIs (mainly acarbose) are poorly absorbed after oral administration.^8^ They work mainly in the small intestine to delay carbohydrate absorption by competitively inhibiting enzymes that convert complex nonabsorbable carbohydrates into simple absorbable carbohydrates.^24^ Therefore, AGIs do not exert pharmacological effects in the body and cannot cross the blood–brain barrier to reach the brain.^8^ New use of TZDs or AGIs was defined as the first prescription of a drug from either class, with no fill of TZDs and AGIs in a baseline washout period of 6 months. The date of the first prescription was defined as the index date. Drug prescriptions were identified using Anatomical Therapeutic Chemical (ATC) system codes. TZDs (ATC A10BG) contained pioglitazone and rosiglitazone, and AGIs (ATC A10BF) included acarbose, miglitol, and voglibose in the study population (Table S1).

Participants aged under 18 years old were excluded from the cohort. We also excluded participants who received combination treatment of TZDs and AGIs at the index date and who had received a diagnosis of any dementia before the index date. In order to ensure that participants actually received treatment of TZDs or AGIs, we further excluded patients who had no consecutive prescriptions of these drugs within 6 months of the index date.

### 2.2 Outcome, follow‐up, and covariates

The primary outcome was all‐cause dementia defined according to the *International Classification of Diseases, 10th Revision* (ICD‐10) codes F00‐F03 and G30. An incident dementia case was defined by using diagnosis records and drug prescriptions: (a) having at least two consecutive diagnosis codes or descriptions of F00‐F03 or G30, or (b) having at least one dementia diagnosis record and prescription record of antidementia drugs (drugs coded as N06D and sodium oligomannate, Table S2). The date of the first diagnosis was defined as the outcome date. The primary analysis was to emulate an intention‐to‐treat analysis to assess the any‐exposure effects of TZD use on dementia risk. Participants were followed up from the index date until the first occurrence of the following events: diagnosis of any dementia, death, last medical record in the database, or the end of the study period (31 December 2021).

Covariates were measured in the baseline washout period and included demographic characteristics (age, sex, and education level); behavior and lifestyle (smoking, drinking, and regular exercise); duration of T2DM; comorbidities measured as Charlson comorbidity index (CCI),^25^ which was calculated according to 16 kinds of diseases (Table S3); co‐use of prescription drugs, including antidiabetic drugs except TZDs and AGIs (insulins, metformin, sulfonylureas, glinides, and other oral glucose lowering agents) and commonly used medications for metabolic and cardiovascular diseases (diuretics, beta‐blocking agents, calcium channel blockers, angiotensin‐converting enzyme inhibitors, angiotensin receptor blockers, and aspirin, lipid modifying agents, and proton‐pump inhibitors). Moreover, measures of diabetes severity including FPG and HbA1c, blood lipid level, blood pressure, body mass index (BMI), and healthcare utilization (hospitalizations and outpatient visits) were also included.

### 2.3 Statistical analyses

For baseline characteristics, we calculated and reported mean and standard deviation for continuous covariates and frequency and percentage for categorical variables. Standardized mean difference (SMD) was used for comparisons between users of TZDs and AGIs. An SMD less than 0.1 indicated that covariates were effectively balanced between groups of the two drugs.^26^

In the primary analysis, we used a Cox regression model with inverse probability of treatment weighting (IPTW) to estimate the hazard ratio (HR) with the 95% confidence interval (CI) for the association between TZD use and risk of dementia. A logistic regression model was fitted to estimate a stabilized IPTW, of which the denominator was the probability of receiving the treatment that the patient actually received condition on all measured covariates, and the numerator was the marginal probability of TZD or AGI use in the overall sample. All continuous variables such as FPG and HbA1c were modeled as a restricted cubic spline with knots at 5th, 25th, 50th, 75th, and 95th percentiles. The year of index date was also included in the model for IPTW to adjust for changes in prescribing patterns over time. In the final weighted models, stabilized IPTW was truncated at the first and 99th percentiles for mitigating impacts of extreme weights. The mean weight of the IPTW was 0.99 (SD 0.18), and the median was 0.97 (interquartile range [IQR], 0.91–1.05), indicating no obvious violation of the positivity assumption, which requires that no subject can have probability zero or one of being treated. In addition, results of crude analyses without adjusting any potential confounding and multivariate regressions were also provided for comparison. We applied multiple imputation for imputing missing data using the full conditional specification method with five imputations according to the quadratic rule recommended by von Hippel.^27^ The proportional hazards assumption was tested using the Schoenfeld residuals method and plots of the log of negative log of survivor function. No obvious violation of this assumption was found. Robust variance was applied for estimating the 95% CIs of HRs when weighted analyses were performed.

### 2.4 Secondary and subgroup analyses

Because several studies indicated that pioglitazone and rosiglitazone may have different effects on dementia,^14^, ^15^ we separately analyzed the associations between incidence of all‐cause dementia and use of pioglitazone and rosiglitazone respectively. We further assessed the effects of TZD use on different types of dementia, which were classified according to the ICD‐10 codes: Alzheimer's disease (ICD‐10 codes F00 and G30), vascular dementia (ICD‐10 code F01), and all other dementia (ICD‐10 codes F02 and F03). We next examined the association between TZD use and incidence of all‐cause dementia within different subgroups for checking potential interactions: age (≤60 and >60 years), sex (female and male), CCI (0 and ≥1), smoking and drinking behavior, FPG (≤7 and >7 mmol/L), HbA1c (≤7% and >7%), BMI (≤24 and >24 kg/m^2^), and duration of diabetes at the index date (≤2.5 and >2.5 years).

### 2.5 Sensitivity analyses

Multiple sensitivity analyses were performed to examine the robustness of the results. First, we emulated a per‐protocol (PP) analysis, in which participants were further censored upon discontinuing the initial drug in addition to the censoring reasons in the primary analysis. This analysis examined the effects of sustained use of TZDs on dementia risk. Treatment discontinuation was defined as no further prescription of the initial drug within 6 months of the previous prescription. This time‐varying artificial censoring could induce selection bias,^28^ for which we applied a marginal structural model with IPTW and time‐varying inverse probability of censoring weighting (IPCW). We fitted a pooled logistic model to estimate the IPCW, which was the inverse of the probability of remaining uncensored at each follow‐up conditioned on time‐invariant and time‐varying confounders that included all covariates listed above except demographic characteristics, behavior and lifestyle, duration of T2DM, and year of index date. A 6‐month interval was used for assessing time‐varying exposure and covariates at the beginning of each new period in the PP analysis.

Second, alternative washout periods of 12, 18, and 24 months were applied for defining new users of TZDs and AGIs. Third, unstabilized and untruncated stabilized IPTW were applied to assess the impacts of extreme weights. Fourth, the Fine‐Gray subdistribution hazard model was fitted to check possible competing risk of all‐cause mortality. Fifth, the study participants were restricted to T2DM patients aged over 40 years and 65 years at the index date, respectively. This could help exclude cases of early‐onset dementia since young people rarely develop this disease. Sixth, we used several alternative definitions of incident dementia as (a) having at least two diagnoses of ICD‐10 codes of F00‐F03 or G30; (b) having over two diagnoses of any dementia and prescriptions of antidementia agents (Table S2) after the first diagnosis; (c) having a consecutive diagnosis of dementia or prescription of antidementia drugs within 1 year of the first diagnosis of dementia; and (d) having any diagnosis of dementia. Seventh, we excluded T2DM patients who received any antidiabetic drugs in the baseline washout period and evaluated the effects of monotony therapy of TZDs. Eighth, given that metformin has been shown to be associated with lower risk of dementia with a dose–response relationship,^29^ we conducted a stratification analysis using tertiles of the cumulative duration of metformin treatment before the index date. Finally, a period after the index date was excluded for adjusting potential latency time and follow‐up were begun at the end of the latency time. We did series of this kind of analysis by requiring successively longer minimum time of follow‐up by adding 1 month to each analysis, up to a maximum of 24 months. This kind of analysis could also help adjust unmeasured confounding by undiagnosed disease.^28^

Data extraction and management were conducted using Hive and all statistical analyses were performed using SAS 9.4 (SAS Institute Inc, Cary, NC, USA).

## 3 RESULTS

**FIGURE 1:**
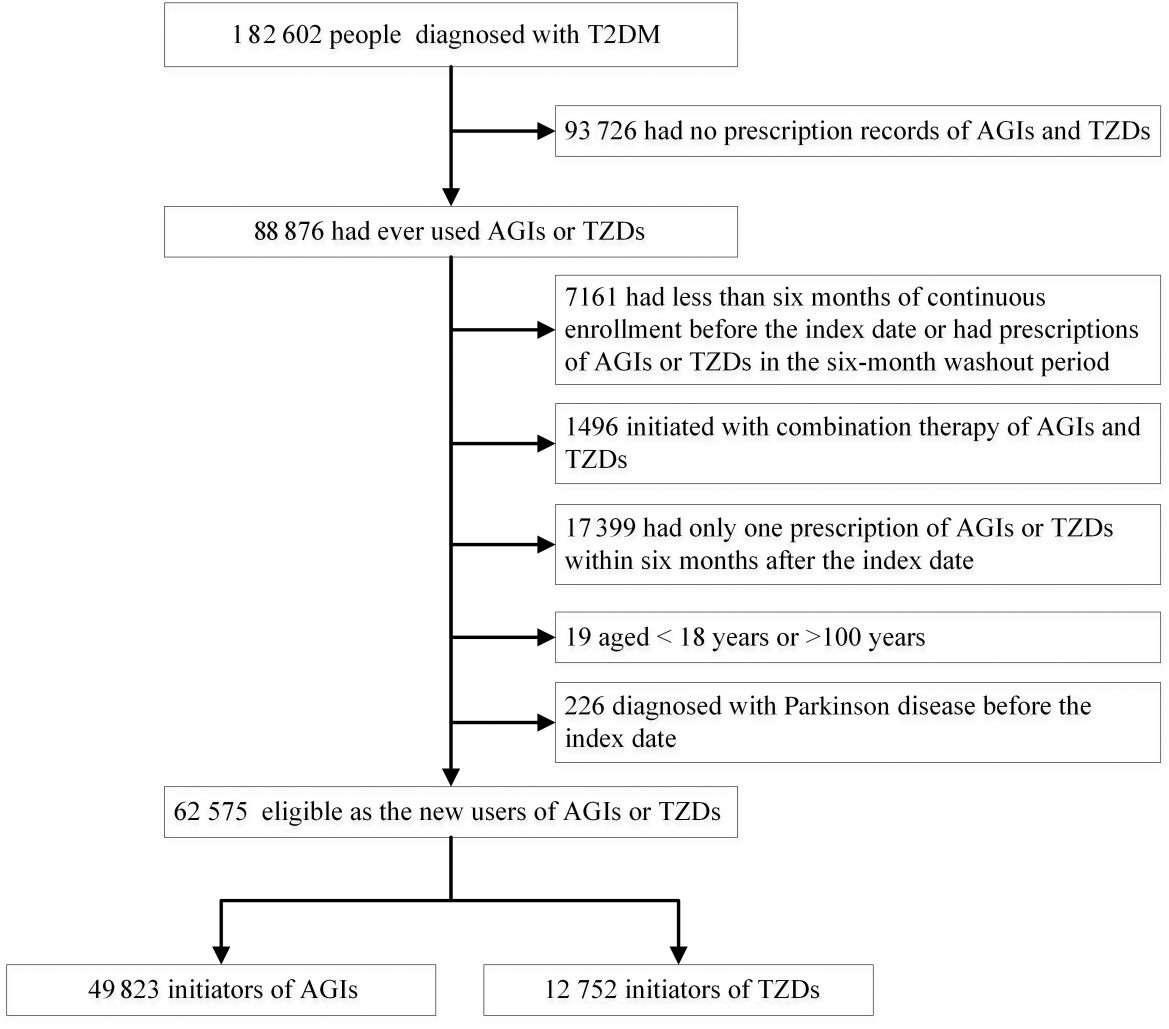
Flow chart of participants in the study cohort. AGIs, alpha glucosidase inhibitors; T2DM, type 2 diabetes mellitus; TZDs, thiazolidinediones.

The final cohort included 49 823 new users of AGIs and 12 752 new users of TZDs (Figure 1), with a median follow‐up time of 5.4 (IQR, 2.6 ~ 8.7) and 6.1 (IQR, 3.1 ~ 8.9) years, respectively. Among TZD users in the unweighted cohort, 47.3% (6030/12 575) were >60 years old and 10.4% (1322/12 575) had an education of senior high school or higher, both of which were lower than those in AGI users (Table 1). However, new users of TZDs had higher BMI and were more likely to receive treatment of metformin and sulfonylureas. In addition, TZD users were less likely to be hospitalized and receive insulin treatment in the baseline period. Nevertheless, after weighting the cohort using inverse probability of treatment, all baseline characteristics were effectively balanced between new users of AGIs and TZDs (SMD < 0.1 for all covariates).

**TABLE 1:** Baseline characteristics of new users of thiazolidinediones or alpha glucosidase inhibitors in the Yinzhou region^a^

	Unweighted population			Weighted population		
Characteristics	AGI users	TZD users	SMD	AGI users	TZD users	SMD
Age, years	61.1 (12.30)	59.0 (11.59)	0.173	60.7 (12.10)	60.5 (12.04)	0.042
Age > 60 years	26 894 (54.0)	6030 (47.3)	0.134	26 236 (52.6)	6292 (50.8)	0.016
Years of T2DM	4.3 (5.02)	3.9 (4.23)	0.086	4.2 (4.85)	4.3 (4.85)	0.027
Years of T2DM >2.5 years	25 477 (51.1)	6618 (51.9)	0.015	25 538 (51.2)	6307 (50.9)	0.006
**Education level**						
Senior high school or higher	7266 (14.6)	1322 (10.4)	0.128	6838 (13.7)	1627 (13.1)	0.017
Junior high school	13 995 (28.1)	3571 (28.0)	0.002	13 985 (28.1)	3471 (28.0)	0.001
Primary school	16 861 (33.8)	4961 (38.9)	0.105	17 377 (34.9)	4373 (35.3)	0.010
Others	11 701 (23.5)	2898 (22.7)	0.018	11 638 (23.4)	2909 (23.5)	0.003
Sex (female)	23 547 (47.3)	6337 (49.7)	0.049	23 813 (47.8)	5997 (48.4)	0.011
Smoking	15 273 (30.7)	3912 (30.7)	0.003	15 288 (30.7)	3824 (30.9)	0.003
Drinking	17 176 (34.5)	4269 (33.5)	0.021	17 083 (34.3)	4225 (34.1)	0.002
**Sport frequency**						
>4 d/w	5744 (11.5)	1839 (14.4)	0.086	6046 (12.1)	1549 (12.5)	0.012
1 ~ 3 d/w	9605 (19.3)	2585 (20.3)	0.025	9712 (19.5)	2421 (19.6)	0.002
<1 d/w	34 474 (69.2)	8328 (65.3)	0.083	34 080 (68.4)	8410 (67.9)	0.010
**Medication use**						
Insulins	4495 (9.0)	660 (5.2)	0.150	4109 (8.2)	928 (7.5)	0.016
Metformin	13 405 (26.9)	4866 (38.2)	0.242	14 582 (29.3)	3789 (30.6)	0.028
Sulfonylureas	14 523 (29.1)	5375 (42.2)	0.274	15 863 (31.8)	4069 (32.9)	0.015
Glinides	3154 (6.3)	947 (7.4)	0.043	3269 (6.6)	821 (6.6)	0.005
Other antidiabetics	1466 (2.9)	415 (3.3)	0.018	1506 (3.0)	397 (3.2)	0.016
PPI	6794 (13.6)	1973 (15.5)	0.052	7002 (14.0)	1783 (14.4)	0.020
ACEI	3465 (7.0)	983 (7.7)	0.029	3548 (7.1)	893 (7.2)	0.002
ARB	13 931 (28.0)	4138 (32.4)	0.098	14 395 (28.9)	3613 (29.2)	0.011
Aspirin	4805 (9.6)	1115 (8.7)	0.031	4717 (9.5)	1158 (9.4)	0.012
Diuretics	7628 (15.3)	2165 (17.0)	0.045	7797 (15.6)	1925 (15.6)	0.002
Beta blocking agents	4745 (9.5)	1149 (9.0)	0.018	4696 (9.4)	1148 (9.3)	0.001
Calcium channel blockers	14 147 (28.4)	3957 (31.0)	0.058	14 424 (28.9)	3597 (29.1)	0.005
Statins	7006 (14.1)	2002 (15.7)	0.046	7176 (14.4)	1774 (14.3)	0.001
Other lipid modifying agents	1281 (2.6)	406 (3.2)	0.037	1349 (2.7)	348 (2.8)	0.008
**CCI**						
0	35 731 (71.7)	9451 (74.1)	0.054	35 978 (72.2)	9002 (72.7)	0.012
1	7801 (15.7)	2081 (16.3)	0.018	7871 (15.8)	1948 (15.7)	0.002
≥2	6291 (12.6)	1220 (9.6)	0.098	5988 (12.0)	1430 (11.6)	0.014
BMI (kg/m2)	23.8 (2.97)	24.3 (3.00)	0.161	23.9 (2.97)	23.9 (2.94)	0.024
DBP (mmHg)	78.0 (6.03)	78.3 (6.02)	0.049	78.1 (5.97)	78.1 (5.94)	0.009
SBP (mmHg)	128.7 (9.25)	128.9 (9.12)	0.017	128.7 (9.13)	128.7 (9.06)	0.002
FPG (mmol/L)	7.3 (2.06)	7.3 (1.98)	0.003	7.3 (2.03)	7.3 (2.02)	0.003
HbA1c (%)	7.6 (1.91)	7.6 (1.87)	0.049	7.6 (1.90)	7.6 (1.89)	0.005
HDLC (mmol/L)	1.2 (0.32)	1.2 (0.31)	0.045	1.2 (0.32)	1.2 (0.31)	0.010
LDLC (mmol/L)	2.7 (0.81)	2.7 (0.81)	0.073	2.7 (0.81)	2.7 (0.81)	0.016
**Outpatient visits**						
0	12 194 (24.5)	2275 (17.8)	0.163	11 522 (23.1)	2822 (22.8)	0.008
1 ~ 6	19 552 (39.2)	4902 (38.4)	0.016	19 458 (39.0)	4800 (38.8)	0.006
7 ~ 12	10 200 (20.5)	3221 (25.3)	0.114	10 682 (21.4)	2668 (21.6)	0.003
>12	7877 (15.8)	2354 (18.5)	0.070	8176 (16.4)	2090 (16.9)	0.013
**Inpatient admissions**						
0	45 554 (91.4)	12 095 (94.8)	0.135	45 911 (92.1)	11 482 (92.7)	0.024
1	3477 (7.0)	579 (4.5)	0.105	3233 (6.5)	767 (6.2)	0.012
≥2	792 (1.6)	78 (0.6)	0.094	693 (1.4)	131 (1.1)	0.030
**Year of index date**						
2009	1855 (3.7)	325 (2.5)	0.067	1740 (3.5)	442 (3.6)	0.004
2010	4071 (8.2)	1131 (8.9)	0.025	4151 (8.3)	1065 (8.6)	0.010
2011	5068 (10.2)	1417 (11.1)	0.030	5162 (10.4)	1284 (10.4)	0.001
2012	5119 (10.3)	1361 (10.7)	0.013	5158 (10.4)	1279 (10.3)	0.001
2013	4139 (8.3)	1252 (9.8)	0.053	4292 (8.6)	1075 (8.7)	0.003
2014	3512 (7.0)	1181 (9.3)	0.081	3747 (7.5)	973 (7.9)	0.013
2015	3573 (7.2)	1014 (8.0)	0.030	3643 (7.3)	885 (7.1)	0.006
2016	3650 (7.3)	1041 (8.2)	0.031	3737 (7.5)	939 (7.6)	0.003
2017	4446 (8.9)	1053 (8.3)	0.024	4379 (8.8)	1094 (8.8)	0.002
2018	3880 (7.8)	736 (5.8)	0.080	3673 (7.4)	876 (7.1)	0.011
2019	3947 (7.9)	624 (4.9)	0.124	3637 (7.3)	829 (6.7)	0.024
2020	3408 (6.8)	713 (5.6)	0.052	3283 (6.6)	811 (6.5)	0.002
2021	3155 (6.3)	904 (7.1)	0.030	3237 (6.5)	828 (6.7)	0.008

Abbreviations: ACEI, angiotensin‐converting enzyme inhibitors; AGIs, alpha glucosidase inhibitors; ARB, angiotensin receptor blockers; BMI, body mass index; CCI, Charlson comorbidity index; DBP, diastolic blood pressure; FPG, fasting plasma glucose; HbA1c, glycated hemoglobin; HDLC, high‐density lipoprotein cholesterol; LDLC, low‐density lipoprotein cholesterol; PPI, proton‐pump inhibitors; SBP, systolic blood pressure; SMD, standardized mean difference; T2DM, type 2 diabetes mellitus; TZDs, thiazolidinediones.

^a^ For continuous variables, the values are means (standard deviation); for categorical variables the values are frequencies (percentage).

**FIGURE 2:**
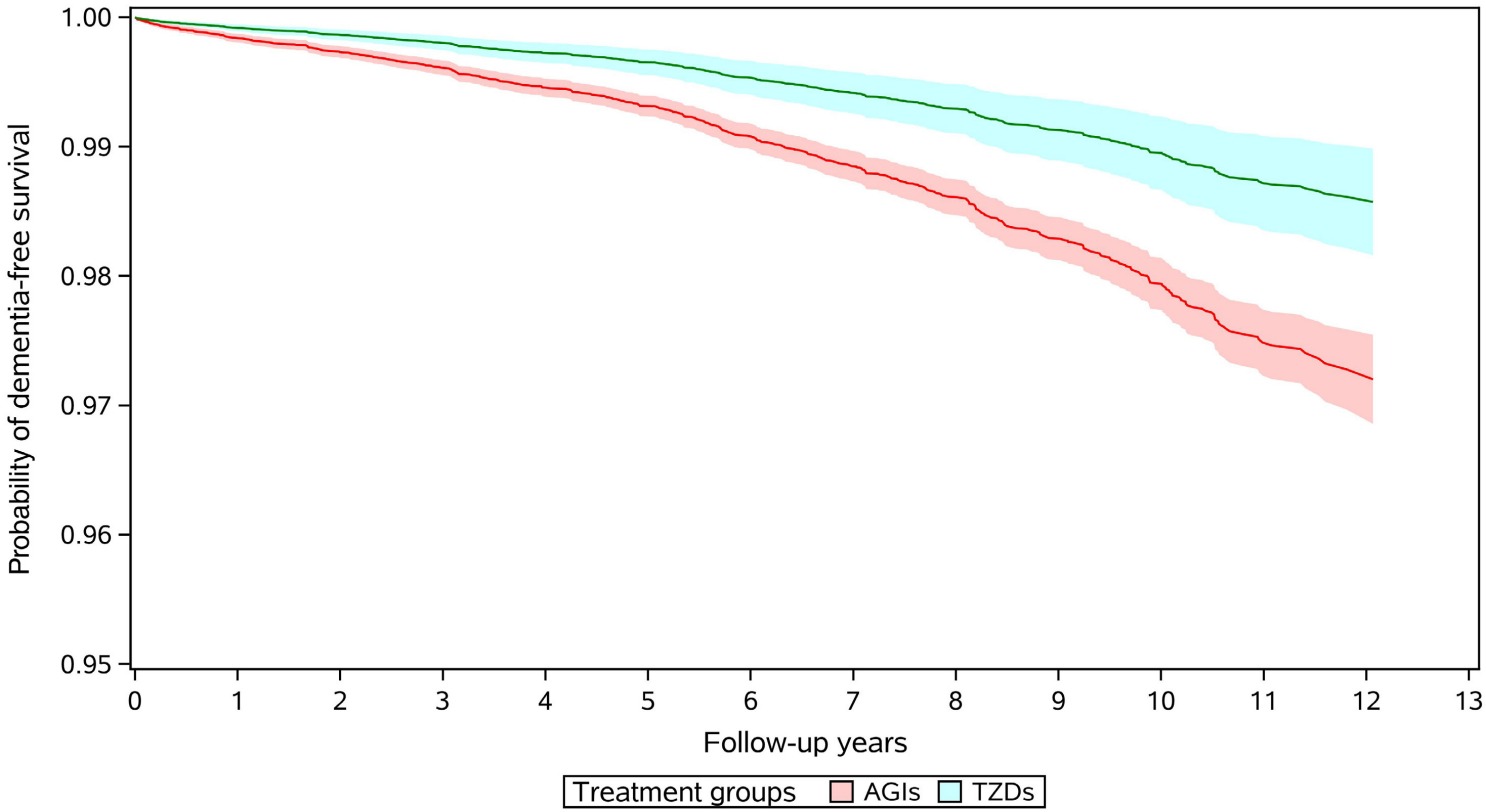
IPTW weighted survival curves of the users of TZDs and AGIs. AGIs, alpha glucosidase inhibitors; IPTW, inverse probability of treatment weighting; TZDs, thiazolidinediones.

A total of 613 incident dementia cases occurred during follow‐up in the primary analysis, of them 463 cases were Alzheimer's disease, 46 were vascular dementia, and 104 were other types of dementia. The incidence of all‐cause dementia was 195.7 per 100 000 person‐years for AGI users and 78.2 per 100 000 person‐years in TZD users respectively. Compared to use of AGIs, the crude analysis indicated a strong protective effect of TZD use against dementia with a HR of 0.40 (95% CI, 0.31–0.52) (Table 2), and multivariate regression model presented a HR of 0.57 (95% CI, 0.43–0.74). After adjusting for potential confounders using stabilized IPTW, use of TZDs was still associated with a 49% reduction in incidence of dementia (HR 0.51, 95% CI, 0.38–0.67). Figure 2 gives the IPTW weighted survival curves of users of AGIs and TZDs. Series analyses in different subpopulations of T2DM patients found similar inverse association between TZD use and incidence of dementia, with HRs ranging from 0.31 (95% CI, 0.18–0.53) to 0.67 (95% CI, 0.47–0.95). No significant interaction was found in all subgroup analyses (Table 2). In the analyses for different kinds of TZDs, rosiglitazone and pioglitazone were associated with the risk of dementia with a HR of 0.31 (95% CI, 0.11–0.89) and 0.52 (95% CI, 0.39–0.69), respectively (Table S4). For different types of dementia, use of TZDs was associated with a 56% decrease in the risk of Alzheimer's disease (HR 0.44; 95% CI, 0.31–0.62), 43% decrease in the risk of vascular dementia (HR 0.57; 95% CI, 0.23–1.42), and 20% decrease in the incidence of other dementias (HR 0.80; 95% CI, 0.45–1.44), respectively (Table S5).

**TABLE 2:** Association between TZD use and incidence of dementia, results of the primary and subgroup analyses

	AGI users		TZD users			HR (95% CI)	
Subgroups	Cases/Person years	Incidence (/100 000 PY)	Cases/Person years	Incidence (/100 000 PY)	Crude analysis	Multivariate regression	IPTW model
**Overall**	553/282 558	195.7	60/76 748	78.2	0.40 (0.31–0.52)	0.57 (0.43–0.74)	0.51 (0.38–0.67)
**Age group**							
≤60 years	57/134 777	42.3	8/414 30	19.3	0.46 (0.22–0.97)	0.48 (0.23–1.03)	0.57 (0.26–1.24)
>60 years	496/147 781	335.6	52/353 19	147.2	0.44 (0.33–0.58)	0.58 (0.43–0.78)	0.51 (0.38–0.70)
**Sex**							
Male	248/142 980	173.5	28/374 49	74.8	0.43 (0.29–0.63)	0.66 (0.44–0.98)	0.52 (0.34–0.79)
Female	305/139 578	218.5	32/392 99	81.4	0.37 (0.26–0.54)	0.50 (0.34–0.73)	0.50 (0.34–0.73)
**Years of T2DM**							
≤2.5 years	191/139 454	137	18/387 20	46.5	0.34 (0.21–0.55)	0.48 (0.29–0.78)	0.40 (0.24–0.67)
>2.5 years	362/143 103	253	42/380 28	110.4	0.44 (0.32–0.61)	0.62 (0.45–0.86)	0.58 (0.41–0.82)
**Drinking**							
No	380/184 678	205.8	37/510 92	72.4	0.35 (0.25–0.49)	0.49 (0.34–0.68)	0.48 (0.33–0.68)
Yes	173/978 79	176.7	23/256 56	89.6	0.51 (0.33–0.78)	0.81 (0.52–1.28)	0.57 (0.36–0.90)
**Smoking**							
No	458/200 062	228.9	48/544 46	88.2	0.38 (0.28–0.52)	0.54 (0.40–0.74)	0.50 (0.36–0.68)
Yes	95/824 96	115.2	12/223 02	53.8	0.47 (0.26–0.86)	0.66 (0.35–1.22)	0.55 (0.29–1.05)
**BMI**							
≤24 kg/m^2^	334/155 044	215.4	33/371 94	88.7	0.42 (0.28–0.62)	0.58 (0.38–0.88)	0.55 (0.35–0.84)
>24 kg/m^2^	219/127 513	171.7	27/395 54	68.3	0.39 (0.24–0.63)	0.54 (0.33–0.89)	0.44 (0.24–0.81)
**CCI**							
0	386/221 188	174.5	42/612 70	68.5	0.39 (0.28–0.54)	0.55 (0.40–0.76)	0.48 (0.34–0.67)
>0	167/613 70	272.1	18/154 79	116.3	0.43 (0.26–0.70)	0.61 (0.37–1.00)	0.58 (0.35–0.96)
**FPG**							
≤7 mmol/L	301/151 185	199.1	42/404 45	103.8	0.52 (0.37–0.73)	0.70 (0.49–1.00)	0.67 (0.47–0.95)
>7 mmol/L	252/131 373	191.8	18/363 04	49.6	0.26 (0.15–0.44)	0.39 (0.23–0.67)	0.31 (0.18–0.53)
**HbA1c**							
≤7%	211/112 096	188.2	28/314 37	89.1	0.48 (0.26–0.87)	0.66 (0.36–1.22)	0.57 (0.31–1.03)
>7%	342/170 461	200.6	32/453 11	70.6	0.34 (0.21–0.57)	0.50 (0.30–0.84)	0.46 (0.28–0.76)

Abbreviations: AGIs: alpha glucosidase inhibitors; BMI, body mass index; CCI, Charlson comorbidity index; CI, confidence interval; FPG, fasting plasma glucose; HbA1c, glycated hemoglobin; HR, hazard ratio; IPTW, inverse probability of treatment weighting; PY, person‐years; T2DM, type 2 diabetes mellitus; TZDs, thiazolidinediones.

In the PP analyses, participants were censored when discontinuing the initial treatment, resulting in a mean follow‐up of 2.2 years. Crude and IPTW models showed a HR of 0.22 (95% CI, 0.11–0.43) and 0.30 (95% CI, 0.15–0.61) respectively (Table 3). Moreover, the IPCW model resulted in a consistent HR, which was 0.35 (95% CI, 0.16–0.74) after adjusting the potential selection bias due to artificial censoring. All sensitivity analyses were consistent (Table 3). First, comparison of new users of both drugs using a washout period of 1 year also indicated that the hazard of dementia in TZD users were 0.51 (95% CI, 0.39–0.67) times that of users of AGIs. Further results of washout periods of 18 and 24 months were consistent. Second, the weighted model using untruncated IPTW presented a HR of 0.52 (95% CI, 0.39–0.69). Third, competing risk due to all‐cause mortality did not show substantial impact and the subdistribution hazard model resulted in a HR of 0.51 (95% CI, 0.39–0.66). Fourth, when participants were restricted to T2DM patients aged ≥40 years or ≥65 years, a HR of 0.51 (95% CI, 0.38–0.68) and 0.50 (95% CI, 0.36–0.70) was observed respectively. Fifth, different definitions of incident dementia yielded HRs ranging from 0.45 (95% CI, 0.31–0.65) to 0.61(95% CI, 0.50–0.75). Sixth, in the subcohort of participants who initiated monotony therapy of TZDs, the incidence of dementia was reduced by 48% (HR 0.52; 95% CI, 0.35–0.79). Seventh, in participants receiving no treatment of metformin before the index date, TZD use was associated with dementia risk with a HR of 0.49 (95% CI, 0.31–0.76). Results in different stratification of cumulative duration of metformin treatment were generally consistent. Finally, HR estimates were robust across potential latency periods of different length, with the minimum HR of 0.51 (95% CI, 0.39–0.66) and maximum HR of 0.55 (95% CI, 0.41–0.73) respectively (Figure S2).

**TABLE 3:** Sensitivity analyses for the association between TZD use and dementia incidence

Sensitivity analyses	AGI users		TZD users		
	Cases/person years	Incidence (/100 000 PY)	Cases/person years	Incidence (/100 000 PY)	HR (95% CI)
**Per‐protocol analyses**					
Crude analysis	204/112 155	181.9	9/22 762	39.5	0.22 (0.11–0.43)
IPTW model	204/112 155	181.9	9/22 762	39.5	0.30 (0.15–0.61)
IPTW and IPCW model	204/112155	181.9	9/22 762	39.5	0.35 (0.16–0.74)
**Alternative washout periods**					
12 months	529/268 707	196.9	58/74 378	78.0	0.51 (0.39–0.67)
18 months	496/253 318	195.8	53/71 782	73.8	0.48 (0.36–0.65)
24 months	466/238 389	195.5	46/68 372	67.3	0.45 (0.33–0.62)
**Alternative weighting model**					
Untruncated IPTW	553/282 558	195.7	60/76 748	78.2	0.52 (0.39–0.69)
Unstabilized IPTW	553/282 558	195.7	60/76 748	78.2	0.51 (0.38–0.67)
**Competing risk model**	553/282 558	195.7	60/76 748	78.2	0.51 (0.39–0.66)
**Subpopulation of older age**					
≥40 years	551/272 004	202.6	60/73 375	81.8	0.51 (0.38–0.68)
≥65 years	440/96 685	455.1	41/21 032	194.9	0.50 (0.36–0.70)
**Alternative outcome definitions** ^a^					
Outcome definition 2	544/282 558	192.5	58/76 748	75.6	0.50 (0.38–0.67)
Outcome definition 3	350/282 558	123.9	34/76 748	44.3	0.45 (0.31–0.65)
Outcome definition 4	594/282 558	210.2	68/76 748	88.6	0.52 (0.40–0.67)
Outcome definition 5	859/282 558	304	115/76 748	149.8	0.61 (0.50–0.75)
**Monotony therapy of TZDs or AGIs**	293/154 842	189.2	27/34 207	78.9	0.52 (0.35–0.79)
**Stratified by duration of metformin use**					
No metformin treatment	301/141 317	213.0	24/28 493	84.2	0.49 (0.31–0.76)
< 6 months	87/50 459	172.4	7/14 780	47.4	0.38 (0.18–0.84)
< 2 years	88/52 337	168.1	13/17 834	72.9	0.56 (0.31–1.03)
≥ 2 years	77/38 445	200.3	16/15 642	102.3	0.65 (0.38–1.17)

Abbreviations: AGI, alpha glucosidase inhibitors; CI, confidence interval; HR, hazard ratio; IPCW, inverse probability of censoring weighting; IPTW, inverse probability of treatment weighting; PY, person‐years; TZD, thiazolidinediones.

^**a**^ Outcome definition 2: Dementia defined as having at least two diagnoses of F00‐F03 or G30; Outcome definition 3: Dementia defined as having over two diagnoses of F00‐F03 or G30 and prescriptions of antidementia agents (Anatomical Therapeutic Chemical N06DA or sodium oligomannate) after the first diagnosis; Outcome definition 4: Dementia defined as having a consecutive diagnosis of F00‐F03 or G30 or prescription of antidementia agents within 1 year of the first diagnosis of dementia; Outcome definition 5: Dementia defined as any diagnosis of F00‐F03 or G30.

## 4 DISCUSSION

To the best of our knowledge, this is the first cohort study investigating the association between TZD use and dementia incidence in a mainland Chinese population. This long‐term population‐based cohort study found a strong inverse association between TZD use and incidence of all‐cause dementia, with the risk of developing dementia reduced by nearly half in TZD users compared with users of AGIs. Our results were consistent in various subgroup analyses and sensitivity analyses, suggesting robustness against potential bias.

A large number of preclinical studies in vivo and in vitro have demonstrated the potential protective effects of TZDs on dementia. TZDs can penetrate the blood–brain barrier, especially pioglitazone.^8^ They can activate PPAR‐γ, which are expressed in various regions of the brain, regulating a range of activities that positively influence the pathology of Alzheimer's disease, including reducing insulin resistance, inhibiting inflammation, improving mitochondrial function, and reducing tau and amyloid pathology.^9^, ^30^, ^31^ In addition, in animal models TZDs presented to protect against vascular dementia through actions of antioxidative, antiacetyl cholinesterase, and anti‐inflammation.^32^ This study was in line with these preclinical findings, with our results showing that use of TZDs was associated a reduced incidence of different types of dementia, especially Alzheimer's disease. The insignificant findings in dementias except Alzheimer's disease could be due to the limited number of events, thus more studies are needed to assess the association between TZD use and vascular and other types of dementia.

Our results were supported by several previous studies. A recent large‐scale cohort study conducted in Korea found that in T2DM patients receiving metformin‐based dual oral therapy, use of metformin + TZDs and sulfonylureas + TZDs could significantly reduce the risk of all‐cause dementia by 20% and 4% respectively compared with use of metformin + sulfonylureas.^33^ Variation in the magnitude of HRs could be due to different comparators, as some evidence indicated that metformin and sulfonylurea might also have effects on dementia risk.^8^, ^34^ Lu et al applied a matching strategy to investigate the use of antidiabetic drugs and dementia risk in the Chinese population living in Taiwan.^31^ They found that the HRs of dementia among users of metformin + pioglitazone were 0.56, 0.66, 0.69, and 0.98, respectively, when compared with metformin‐based use of sulfonylureas, acarbose, meglitinide, and insulin.^31^ However, fewer than 2000 patients were included in each analysis in this study, with insignificant results except the analysis comparing TZDs with sulfonylurea.^31^ Furthermore, several studies conducted in Germany,^19^, ^35^ Korea,^16^ and the United States^36^ also presented results consistent with our study. Nevertheless, it should be noted that some studies indicated that only pioglitazone but not rosiglitazone was associated with a lower risk of dementia.^14^, ^15^ In this study both pioglitazone and rosiglitazone presented to be associated with lower risk of all‐cause dementia, which was consistent with a study conducted by Akimoto et al.^36^ Because both Tseng's study and ours included only a small number of rosiglitazone users with a limited number of outcome events, no definitive conclusions could be drawn about the association between the use of rosiglitazone and dementia risk. Therefore, future studies with larger sample sizes are needed to provide further valuable evidence. Other studies did not support the neuroprotective effect of TZDs, among which Cheng et al found that T2DM patients receiving TZD treatment had 1.4 times the risk of developing dementia compared to patients who did not receive TZDs.^13^ Nevertheless, this result of increased risk of dementia in TZD users could be attributed to several kinds of serious bias. First, diabetes‐free participants at baseline were included and followed from a calendar date in the study by Cheng et al.^13^ Then they compared dementia risk between users and nonusers of TZDs in participants who developed T2DM during the follow‐up without considering the time‐varying nature of drug exposure. This kind of design and analysis strategy could result in founding by indication due to comparing treated and nontreated patients and immortal‐time bias because of misclassifying nonexposure time from cohort entry to drug exposure.^37^, ^38^ Similarly, Kim et al followed participants from the date of T2DM diagnosis and defined exposure status according to TZD use in the year after diagnosis.^16^ This look‐forward type of exposure definition could induce selection bias and immortal time bias, artificially causing a longer follow‐up time in the exposure group (5.3 years in patients receiving drug treatment and 3.8 years in the control group), which would lead to underestimation of the outcome incidence in the exposure group.

In clinical trials, a recent phase 3, multicenter randomized, placebo‐controlled trial showed that pioglitazone did not delay the onset of mild cognitive impairment among participants at high risk.^39^ However, this trial excluded diabetes patients at baseline, thus data from this trial may not generalize to T2DM patients because of different causes of cognitive impairment.^40^ In contrast, two meta‐analyses of randomized controlled trials concluded that use of TZD could improve cognitive performance in patients with Alzheimer's disease or mild cognitive impairment.^41^, ^42^ However, high heterogeneity should be noted among these studies due to variation in participants, intervention, follow‐up, and outcome definition.^41^, ^42^ In addition, our study was aimed to investigate the association between TZD use and dementia incidence in T2DM patients and could not provide evidence on whether this drug could modify the progression of dementia after disease onset because the effects of antidiabetic drugs on dementia risk in the context of prevention and treatment may have different mechanisms.^40^

Dementia poses a great economy and disease burden in China. It is estimated that over 15 million people aged 60 years or older in China suffer from dementia,^20^ and the national annual cost on Alzheimer's disease alone exceeds $160 billion, which will approximate $2 trillion in 2050 in China.^43^ Given the lack of effective disease‐modifying drugs for dementia and a substantial proportion of dementia patients undertreated in China,^44^ developing new therapeutic approaches to dementia is critical for reducing the health burden of this disease. Thus our finding of the potential neuroprotective effect of TZDs in T2DM patients might provide some insights into developing effective prevention and control measures to reduce the future burden of dementia.

Our study had several strengths. We applied an ACNU design, in which AGIs that are used in the same stage of T2DM as TZDs in China^23^ were used as the comparator, thus we avoided immortal‐time bias and minimized confounding by indication through adjusting various potential confounders. In addition, we conducted series of sensitivity analyses for the competing risk, latency period, model specification, outcome validation, analysis strategy, and potential confounding by co‐use of antidiabetic drugs. Results were highly consistent across all sensitivity analyses, indicating robustness of our finding against potential bias. This study also had some limitations. First, T2DM patients in this study were from only one municipal district in China, whereas numerous studies indicated that incidence and prevalence of dementia vary greatly in different regions,^44^ thus extrapolation of the findings of this study to other populations should be made with caution. Second, although we considered quite a number of potential confounders in the analyses and ACNU design might further mitigate the bias related to unmeasured confounding,^39^ some potential confounders, such as dietary patterns, were not adjusted due to lack of accurate information in the database. The *E*‐value, which is the minimum strength of association with both the exposure and outcome needed for an unmeasured confounder to explain away the observed association,^45^ was 3.33 for the observed HR of 0.51 for TZD use and incident dementia. However, associations between dementia and commonly modifiable risk factors are unlikely to be of this magnitude.^46^ Third, we did not investigate the relationship between cumulative TZD use and risk of dementia because accurate dosage information of drugs was not recorded in the database. Future studies focusing on the dose–response relationship between cumulative TZD exposure and risk of dementia would provide further valuable evidence. Finally, like the majority of previous observational studies we used a by‐proxy definition of dementia, of which the accurate diagnosis needs special medical equipment and professional personnel.^44^ Therefore, there might be outcome misclassification in our study. To adjust for potential bias caused by outcome misclassification, we applied several different dementia definitions combing diagnosis and prescription records related to this disease. Our sensitivity analyses of various outcome definitions were highly consistent, thus suggesting that there was little difference in outcome misclassification between users of AGIs and TZDs and our results were robust against potential misclassification bias.

## 5 CONCLUSION

Long‐term use of TZD presented a protective effect against dementia incidence in a mainland Chinese T2DM population. This neuroprotective effect might help to develop effective prevention and control measures to reduce the future incidence of dementia in China.

## AUTHOR CONTRIBUTIONS

Houyu Zhao conceived of and designed the work. Hongbo Lin, Peng Shen, and Yexiang Sun acquired the data. Houyu Zhao analyzed the data. Houyu Zhao drafted the manuscript. Lin Zhuo and Siyan Zhan critically revised the manuscript for important intellectual content. Siyan Zhan, Hongbo Lin, and Peng Shen supervised the study. Houyu Zhao and Siyan Zhan obtained the funding. All authors were responsible for the interpretation of the data, and revised, and gave final approval of the manuscript. Siyan Zhan is the guarantor of this work and, as such, had full access to all the data in the study and takes responsibility for the integrity of the data and the accuracy of the data analysis.

## FUNDING INFORMATION

This work was supported by a grant from the China Postdoctoral Science Foundation (grant NO. 2022 M710251) and a grant from the National Natural Science Foundation of China (Grant No. 81973146 and 82204157). The funder had no role in the study design, data collection, data analysis, data interpretation, writing of the report, or decision to submit the article for publication.

## ETHICS APPROVAL

The study was approved by the ethical review board of Peking University Health Science Center (approval number: IRB00001052‐18013‐Exempt). Informed consent was not required owing to the use of anonymized routine data.

## DISCLOSURE

The authors have no conflicts of interest to declare.

## Supporting information


**DATA S1:** Supporting Information.

## Data Availability

The data that support the findings of this study are available from the corresponding author Siyan Zhan but restrictions apply to the availability of these data, which were used under license for the current study and therefore are not publicly available. Data are however available from the authors upon reasonable request and with permission of the Yinzhou District Center for Disease Control and Prevention.
