# Supplementary material for: Thiazolidinedione use is associated with reduced risk of dementia in patients with type 2 diabetes mellitus: A retrospective cohort study
Source: J Diabetes. 2023 Jan 20;15(2):97–109. doi: 10.1111/1753-0407.13352 (PMC9934955; doi:10.1111/1753-0407.13352)
Supplement: Supplementary file 1 — DATA S1: Supporting Information. [file JDB-15-97-s001.pdf]

Thiazolidinedione use is associated with reduced risk of dementia in patients with type 2 diabetes mellitus: A retrospective cohort study

Houyu Zhao[1], Lin Zhuo[2], Yexiang Sun[3], Peng Shen[3], Hongbo Lin[3], Siyan Zhan[1,2,4,*]
[1] Department of Epidemiology and Biostatistics, School of Public Health, Peking University, Beijing, China (Houyu Zhao, Siyan Zhan);
[2] Research Center of Clinical Epidemiology, Peking University Third Hospital, Beijing, China (Lin Zhuo, Siyan Zhan);
[3] Yinzhou District Center for Disease Control and Prevention, Ningbo, China (Peng Shen, Hongbo Lin, Yexiang Sun);
[4] Center for Intelligent Public Health, Institute for Artificial Intelligence, Peking University, Beijing, China (Siyan Zhan).

Table of Contents

Figure S1. Data structure of the YRHCD. 2
Table S1. Alpha glucosidase inhibitors and thiazolidinediones used in the study population 3
Table S2. Anti-dementia drugs used in the study population 4
Table S3. Comorbidities used to define Charlson comorbidity index 5
Table S4. Association between use of different kinds of TZDs and incidence of all-cause dementia 6
Table S5. Association between use of TZDs and incidence of different types of dementia. 7
Figure S2. Association between TZD use and dementia incidence after excluding different length of latency periods. 8

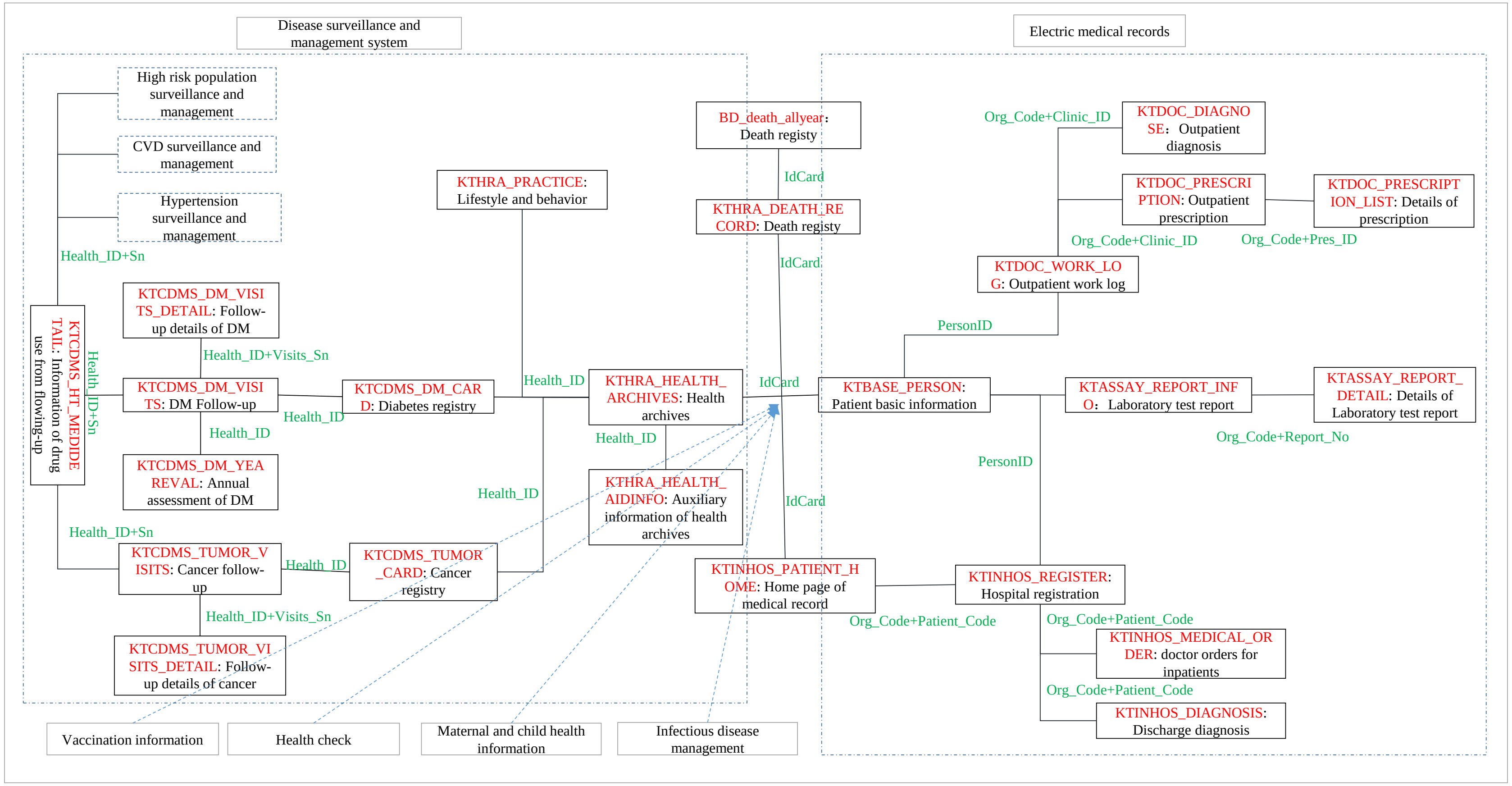

Figure S1. Data structure of the YRHCD.

Relationship of different tables is presented in the figure. Data from the disease surveillance and management and electric medical records were used in this study. Detailed information contained in each table in the figure and more details of other data can be found elsewhere[1, 2].
Red words are table names. Green words are keys for data linking.

Table S1. Alpha glucosidase inhibitors and thiazolidinediones used in the study population

Alpha glucosidase inhibitors				Thiazolidinediones		
**ATC code**	**Drug name**	**N (%)**		**ATC code**	**Drug name**	**N (%)**
A10BF01	Acarbose	2560436(91.4)		A10BG03	Pioglitazone	783201 (94.6)
A10BF02	Miglitol	124506(4.5)		A10BG02	Rosiglitazone	28660(3.5)
A10BF03	Voglibose	115087(4.1)		A10BD05	Metformin and pioglitazone	15614(1.9)
A10BD17	Metformin and acarbose	1(0.0)		A10BD03	Metformin and rosiglitazone	48(0.01)

Table S2. Anti-dementia drugs used in the study population

ATC code	Drug name
N06DA02	Donepezil
N06DA04	Galantamine
N06DA03	Rivastigmine
N06DX01	Memantine
N06DX02	Ginkgo folium
Not available	Sodium oligomannate

Table S3. Comorbidities used to define Charlson comorbidity index

**Comorbidities**[a]	**Defined according to ICD-10 code.**	**Adjustment in the model**
Myocardial infarction	I21.x, I22.x, I25.2, I25.5	For calculating CCI, not included in the final analysis models
Congestive heart failure	I11.0, I13.0, I13.2, I50	Ditto
Peripheral vascular disease	I70, I71, I73.1, I73.8, I73.9, I77.1, I79.0, I792, K55.1, K55.8, K55.9, Z95.8, Z95.9	Ditto
Cerebrovascular disease	G45, G46, H34.0, I60-I69	Ditto
Chronic pulmonary disease	I278, I279, J40-J47, J60-J67, J684, J701, J703	Ditto
Connective tissue disease	M05, M06, M31.5, M32, M33, M34, M35.1, M353, M36.0	Ditto
Ulcer disease	K25-K28	Ditto
Mild liver disease	B18, K70.0-K70.3, K709, K71.3-K71.5, K71.7, K73, K74, K76.0, K76.2-K76.4, K76.8, K76.9, Z94.4	Ditto
Diabetic complication	E10.2-E10.5, E10.7, E11.2-E11.5, E11.7, E12.2-E12.5, E12.7, E13.2-E13.5, E13.7, E14.2-E14.5, E14.7	Ditto
Hemiplegia	G041, G114, G801, G802, G81, G82, G830, G831-G834, G839	Ditto
Diabetes with chronic complication	E102-E105, E107, E112-E115, E117, E122-E125, E127, E132-E135, E137, E142-E145, E147	Ditto
Moderate or severe renal disease	I120, I131, N032-N037, N052-N057, N18, N19, N250, Z49.0-Z49.2, Z940, Z992	Ditto
Any tumor (including lymphoma and leukemia except for malignant neoplasm of skin)	C00-C26, C30-C34, C37-C41, C43, C45-C58, C60-C76, C81-C85, C88, C90-C97	Ditto
Moderate or severe liver disease	I850, I859, I864, I982, K704, K711, K721, K729, K765-K767	Ditto
Metastatic solid tumor	C77-C80	Ditto
HIV/AIDS	B20-B22, B24, Z21	Ditto

[a] Defined according to the 10th version of International Classification of Disease (ICD-10).

Table S4. Association between use of different kinds of TZDs and incidence of all-cause dementia*

	Cases/Person years	Incidence (/100000 PY)	HR (95% CI)		
			Crude analysis	Multivariate regression	IPTW model
**Rosiglitazone use as exposure**					
AGI group	553/282558	195.7	1.00	1.00	1.00
TZD group	4/6835	58.5	0.27 (0.10-0.73)	0.57 (0.21-1.55)	0.31 (0.11 − 0.89)
**Pioglitazone use as exposure**					
AGI group	553/282558	195.7	1.00	1.00	1.00
TZD group	56/69898	80.1	0.41 (0.31-0.54)	0.57 (0.43-0.75)	0.52 (0.39 − 0.69)

*Three individuals received combination treatment of Rosiglitazone and Pioglitazone at baseline were excluded this analysis.

Table S5. Association between use of TZDs and incidence of different types of dementia.

	AGI users		TZD users		HR (95% CI)		
	Cases/Person years	Incidence (/100000 PY)	Cases/Person years	Incidence (/100000 PY)	Crude analysis	Multivariate regression	IPTW model
Alzheimer's disease	424/282558	150.1	39/76748	50.8	0.34 (0.24 − 0.47)	0.50 (0.36 − 0.70)	0.44 (0.31 − 0.62)
Vascular dementia	40/282558	14.2	6/76748	7.8	0.55 (0.23 − 1.29)	0.74 (0.30 − 1.78)	0.57 (0.23 − 1.42)
Other dementias	89/282558	31.5	15/76748	19.5	0.62 (0.36 − 1.07)	0.77 (0.44 − 1.35)	0.80 (0.45 − 1.44)

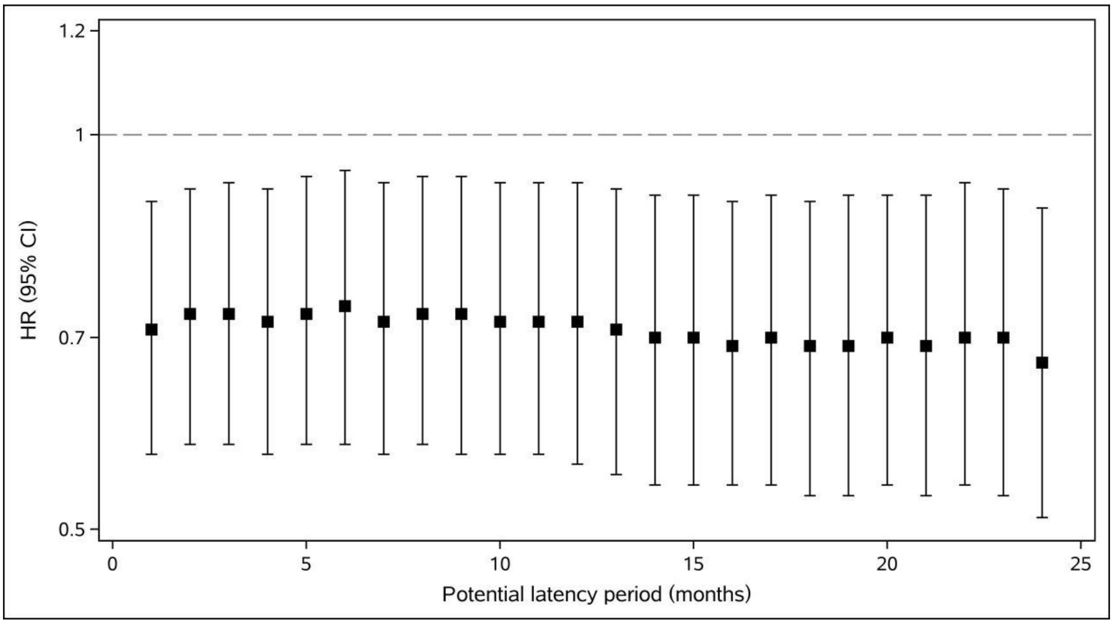

Figure S2. Association between TZD use and dementia incidence after excluding different length of latency periods.

References

1. Lin H, Tang X, Shen P, et al. Using big data to improve cardiovascular care and outcomes in China: a protocol for the CHinese Electronic health Records Research in Yinzhou (CHERRY) Study. *BMJ Open.* 2018;8(2):e19698.

2. Zhao H, Liu Z, Zhuo L, et al. Sulfonylurea and cancer risk among patients with type 2 diabetes: a population-based cohort study. *Front Endocrinol.* 2022;13.
